# Wireless light energy harvesting and communication in a waterproof GaN optoelectronic system

**DOI:** 10.1038/s44172-022-00016-5

**Published:** 2022-07-07

**Authors:** Xumin Gao, Pengzhan Liu, Qingxi Yin, Hao Wang, Jianwei Fu, Fangren Hu, Yuan Jiang, Hongbo Zhu, Yongjin Wang

**Affiliations:** Grünberg Research Centre, Nanjing University of Posts and Telecommunications, Nanjing, 210003 China

**Keywords:** Lasers, LEDs and light sources, Devices for energy harvesting

## Abstract

Wireless technologies can be used to track and observe freely moving animals. InGaN/GaN light-emitting diodes (LEDs) allow for underwater optical wireless communication due to the small water attenuation in the blue-green spectrum region. GaN-based quantum well diodes can also harvest and detect light. Here, we report a monolithic GaN optoelectronic system (MGOS) that integrates an energy harvester, LED and SiO_2_/TiO_2_ distributed Bragg reflector (DBR) into a single chip. The DBR serves as waterproof layer as well as optical filter. The waterproof MGOS can operate in boiling water and ice without external interconnect circuits. The units transform coded information from an external light source into electrical energy and directly activate the LEDs for illumination and relaying light information. We demonstrate that our MGOS chips, when attached to *Carassius auratus* fish freely swimming in a water tank, simultaneously conduct wireless energy harvesting and light communication. Our devices could be useful for tracking, observation and interacting with aquatic animals.

## Abstract (web summary)

Xumin Gao and colleagues report a robust and waterproof monolithic GaN optoelectronic chip that integrates an energy harvester, light emitting diode and Bragg reflector. The units transform external light into electrical energy and directly activate the integrated LEDs even when attached to freely swimming *Carassius auratus* fish.

## Introduction

The rapid development of wearable/implantable optoelectronics for aquatic animals is accompanied by a demand for studying underwater power transfer and communication^1–4^. Although batteries can provide reliable and high power intensity, the disadvantages of their size, weight and replacement restrict their further application in wearable, miniaturized optoelectronic systems in underwater environments. Segev and his team built a waterproof box for wireless electronics to study archerfish behavior and visual processing. However, the freely-swimming fish cannot behave normally because the box is too large^5^. Simultaneous wireless power transfer and communication is a key technology^6–9^. Conventional wireless inductive power transfer for miniaturized optoelectronic systems has been extensively used for optogenetic studies in land living animals^10–17^. Lee and coworkers inserted flexible vertical light-emitting diode (LED) arrays in a living mouse cortex, which were wirelessly powered by using a resonant inductive coupling system^18^. Mickle et al. implanted a miniatured closed-loop optogenetic control system to sense and control bladder function in rats, wherein a wirelessly powered base-station electronic device inside the abdomen causes the LEDs to turn on for illumination^19^. However, despite these efforts, wearable optoelectronics that can perform underwater wireless energy transfer and communication are limited for aquatic animals due to their strong water attenuation in the radio frequency region.

InGaN/GaN LEDs are suitable for underwater wireless light communication because of their relatively small water attenuation in the blue-green spectrum region^20–25^. Recent advances in III-nitride micro-LEDs have also led to the development of next-generation displays and other applications due to their many advantages. These efforts were demonstrated in pioneering work by Dawson and his co-workers^26–31^. In addition to light emission, GaN-based quantum well diodes (QWDs) can convert light into different forms of electricity and signals via the photovoltaic effect, simultaneously exhibiting inherent light detection, emission and energy-harvesting functionalities^32–36^. Multiple GaN QWDs combined into a single chip will simultaneously achieve different functions^37,38^.

Here, we monolithically integrate previously competing detection, emission and energy-harvesting operations into a single chip, thereby forming a monolithic GaN optoelectronic system (MGOS). After depositing SiO_2_/TiO_2_ distributed Bragg reflector (DBR), the MGOS is waterproofed. In addition to waterproof functionality, SiO_2_/TiO_2_ DBR can separate the emitted light from the excited ones. The MGOS does not require an additional external circuit because both the harvesting unit and LED are wire connected on the chip. The harvesting unit captures information-coded light from an external light source and converts it into electrical energy and signals. The harvesting unit is then responsible for supplying energy and signals to a monolithically integrated LED, simultaneously achieving both light energy harvesting and communication in harsh environments. Compared with traditional tethered devices, such MGOS that transfers both energy and information wirelessly will be an essential tool to study the neural complexities of animals, which can move freely and express natural behaviors.

## Results

### Profiles of waterproof MGOS

**Fig. 1:**
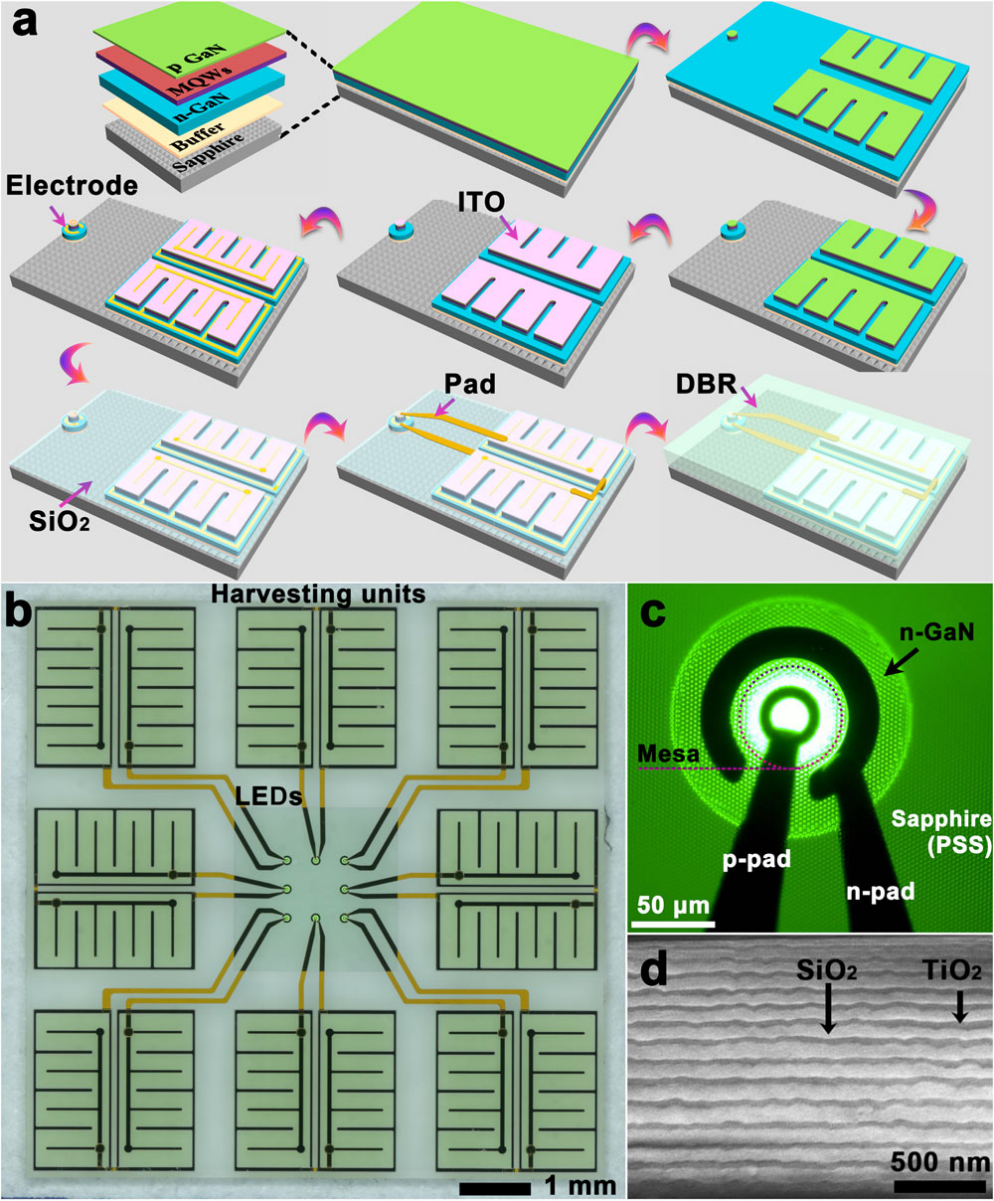
Profiles of waterproof monolithic GaN optoelectronic system (MGOS). **a** Schematic fabrication process of the waterproof MGOS. ITO, indium tin oxide; DBR, distributed Bragg reflector; LED, light emitting diode; PSS, patterned sapphire substrate. **b** Optical microscopy image of the MGOS consisting of eight harvesting units and eight LEDs on a single chip. **c** Monolithically integrated 60 μm-diameter LED. **d** Cross-sectional scanning electron microscope image of the SiO_2_/TiO_2_ DBR.

Combining the harvesting unit, LED and SiO_2_/TiO_2_ DBR leads to a waterproof device architecture. Figure 1a schematically illustrates the fabrication process of the waterproof MGOS, wherein both harvesting units and LEDs share identical InGaN/GaN QW structures. Figure 1b shows an optical microscopy image of a MGOS consisting of eight cells into a single chip. The whole chip is 0.2 mm thick, 6.9 mm wide and 6.9 mm long, and its weight is 42 mg. Individual cell is composed of a harvesting unit and a 60 μm-diameter LED, and can work independently. As shown in Fig. 1c, each harvesting unit interconnects with the LED via metal wires. To achieve high open-circuit voltage (*V*_*oc*_) and consequently power the micro-LED^39^, the two 1 × 2 mm^2^ QWDs connected in a series have merged to form an energy harvester, which will convert coded light into electrical energy and signals through the photovoltaic effect. Figure 1d illustrates a cross-sectional scanning electron microscope image of a 2.28 μm-thick SiO_2_/TiO_2_ DBR that is deposited on the device surface by using optical thin film coater. In order to achieve the desired reflectance spectra, the DBR has an inhomogeneous thickness distribution of the SiO_2_/TiO_2_ pair. The SiO_2_/TiO_2_ DBR has three distinct advantages: (i) it functions as an electrical isolation layer to form a waterproof architecture; (ii) it serves as an optical filter that separates the emitted light from the excited light to pass through the DBR; (iii) it operates as an optical reflector that reflects the incident light back into an upward direction to improve light absorption.

**Fig. 2:**
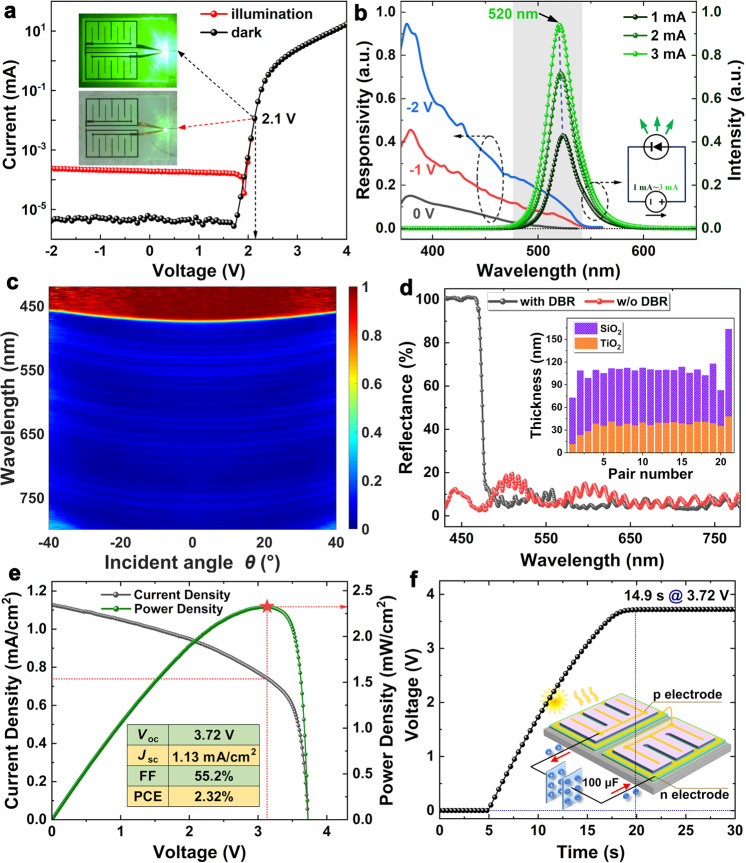
Electrical and optical characteristics of the monolithic GaN optoelectronic system (MGOS) chip. **a** Typical I-V plots with (black curve with circles) and without (red curve with circles) external illumination, where the inset shows the light emission image biased at 2.1 V. **b** Measured electroluminescence and responsivity spectra of the GaN-based quantum well diode. **c** Angle-resolved reflectivity spectra measured from the SiO_2_/TiO_2_ distributed Bragg reflector (DBR) surface. Dark blue indicates the regions with low reflectivity, while dark red denotes the areas with high reflectivity. **d** Comparison of normal reflectivity spectra for device with (black curve with circles) and without (red curve with circles) the SiO_2_/TiO_2_ DBR. **e** Current density (black curve with circles) and power density (green curve with circles) versus voltage characteristics of individual harvesting units under one-sun illumination. *V*_*oc*_, open-circuit voltage; *J*_*sc*_, short-circuit current density; FF, filling factor; PCE, photon conversion efficiency. **f** Time-dependent voltage curves of a 100-μF supercapacitor charged by one harvesting unit, where the inset shows the schematic diagram of charging process.

Figure 2a shows the typical current-voltage (I-V) plots of the LED with/without environmental light, and the inset illustrates the emission image at a forward voltage of 2.1 V. The GaN QWD can absorb light photons to produce a photocurrent. The electroluminescence (EL) spectra of the QWD are plotted as a function of injection current in Fig. 2b. When the device operates as a light-emitting device, the light emission increases, and the dominant EL peak exhibits a slight blue shift from 523.9 to 520.2 nm with increasing injection current from 1 to 3 mA. On the other hand, the device can harvest energy from environmental light when it operates as an energy harvester. Furthermore, spectral emission-responsivity overlap endows the QWD with the capability to harvest, detect and modulate the light emitted by the device sharing an identical quantum well structure. Based on this intriguing spectral overlap, a variety of sophisticated GaN photonic circuits have been studied^40–43^, opening feasible routes to monolithically integrate GaN photonics with electronics on a tiny chip. Figure 2c shows the angle-resolved reflectivity spectra of the chip measured from the SiO_2_/TiO_2_ DBR surface. Dark blue indicates the regions with low reflectivity, while dark red denotes the areas with high reflectivity. The SiO_2_/TiO_2_ DBR exhibits uniform reflectivity for the incident angle from −40^o^ to 40^o^. Compared with the device without the SiO_2_/TiO_2_ DBR, the device allows its light emission but suppresses shorter-wavelength light traveling through the DBR, as shown in Fig. 2d. Figure 2e shows that one harvesting unit can generate a *V*_*oc*_ of 3.72 V and a short-circuit current density (*J*_*sc*_) of 1.13 mA/cm^2^ with a photon conversion efficiency (PCE) of 2.32% under 1-sun illumination. The generated energy can be stored in a battery or supercapacitor. Figure 2f show that individual harvesting units can charge a 100 μF supercapacitor to a steady-state voltage of 3.72 V in 14.9 s.

### Wireless energy harvesting and light communications

**Fig. 3:**
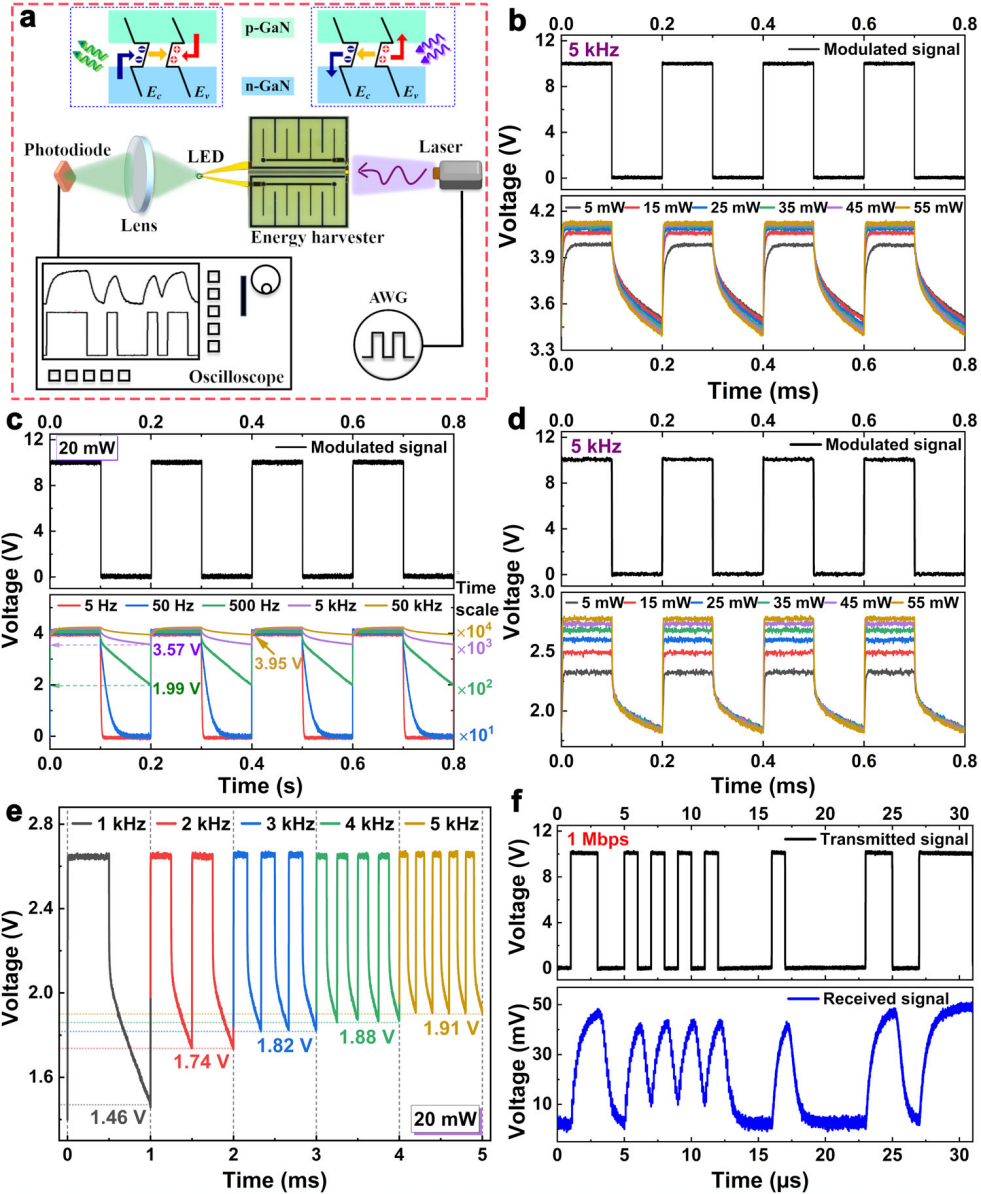
The experimental setup and wireless light communication characterizations. **a** Schematic diagram of a monolithically integrated transmitter remotely powered by light. AWG, arbitrary waveform generator. **b** Steady signal amplitudes of the unit versus the output powers of the 405-nm laser at a modulation frequency of 5 kHz. **c** Decay signal amplitudes of the unit versus the modulation frequencies at a laser output power of 20 mW when the harvesting unit is not connected to the LED. **d** Steady signal amplitudes of the LED versus the output powers of the 405-nm laser at a modulation frequency of 5 kHz. **e** Decay signal amplitudes of the LED versus the modulation frequencies at a laser output power of 20 mW when the harvesting unit is connected with the LED. **f** Comparisons between original and received pseudorandom binary sequence signals at a transmission rate of 1 Mbps.

As schematically illustrated in Fig. 3a, the MGOS can simultaneously achieve wireless energy harvesting and communication through light. The harvesting unit offers the coexistence of light energy-harvesting and light energy-sensing functionalities. Therefore, the unit converts information-coded light into electrical energy when the 405-nm laser pulses its light into coded signals. The harvested energy depends on the illumination power. As shown in Fig. 3b, when it doesn’t interconnect with a monolithically integrated LED, the harvesting unit produces steady signal outputs from 3.99 to 4.12 V at a modulation frequency of 5 kHz as the laser output power increases from 5 to 55 mW. Without external illumination, the harvested energy decays with time. As a result, the unit produces higher decay signal outputs as the modulation frequency increases. Figure 3c illustrates that the decay signal outputs are 1.99, 3.57, and 3.95 V at modulation frequencies of 500, 5000, and 50000 Hz, respectively, and a laser output power of 20 mW. When the harvesting unit is connected to the LED, the encoded power directly turns on the LED, thereby emitting information-coded light for both illumination and relay communication at the same time. The detailed energy harvesting performance is provided in Supplementary Figure 1, see Supplementary Information. As demonstrated in Fig. 3d, the harvesting unit can supply the LED with steady operating voltages from 2.33 to 2.78 V at a modulation frequency of 5 kHz as the laser output power increases from 5 to 55 mW. Figure 3e shows that the decay signal amplitudes of the LED increase from 1.46 to 1.91 V as the modulation frequency increases from 1 to 5 kHz at a laser output power of 20 mW. Furthermore, the external receiver can decode information-coded light from monolithically integrated LEDs for relay wireless light communication. An external photodiode captures this information-coded light and demodulates it to recover electrical signals, which are directly sent to a digital storage oscilloscope without additional preshaping or circuit amplification. Figure 3f compares the original pseudorandom binary sequence signals and the received ones at a data transmission rate of 1 Mbps. The results suggest that the MGOS has the potential for relay wireless light communication. The detailed communication characterization is provided in Supplementary Figs. 2–4, see Supplementary Information.

**Fig. 4:**
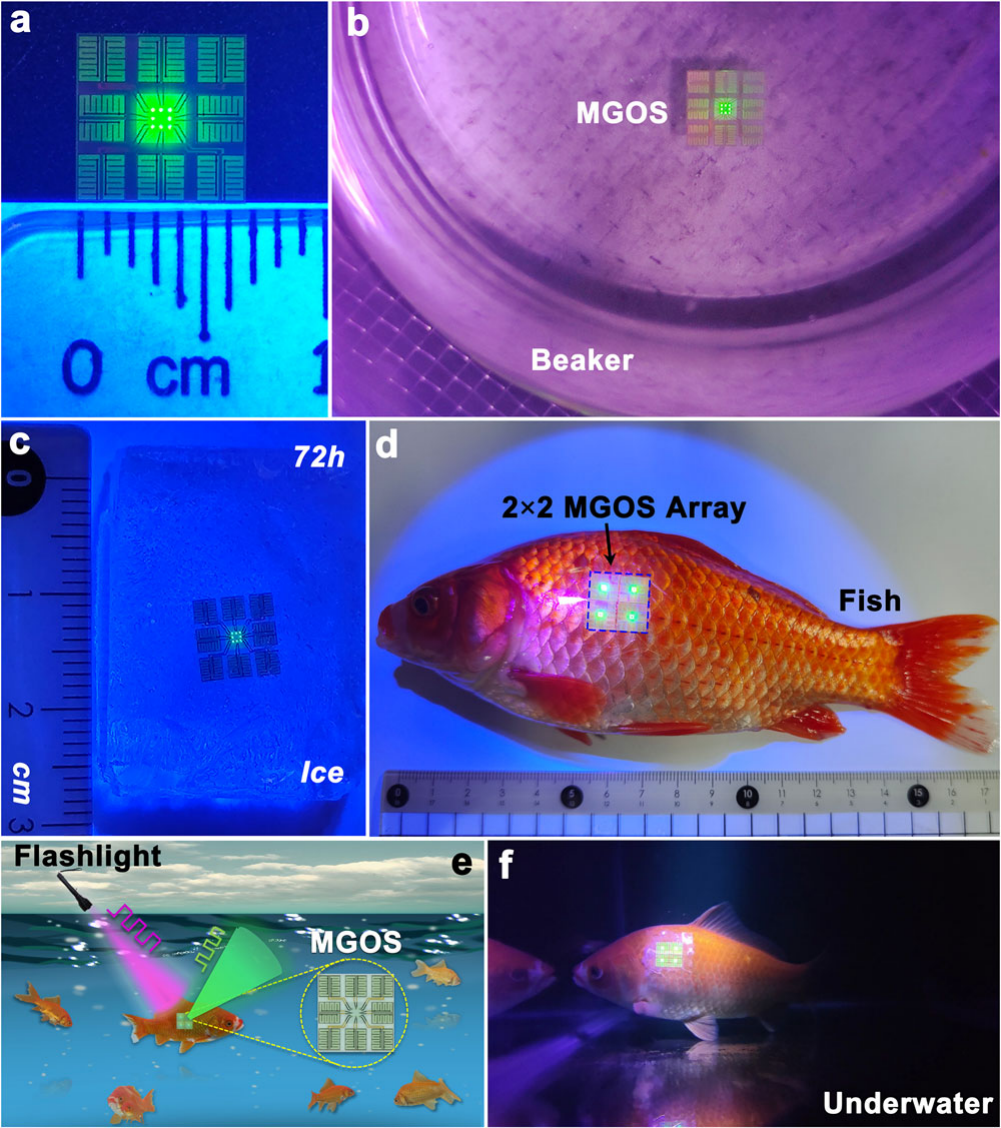
Underwater optical wireless communication system. **a** Picture of an active monolithic GaN optoelectronic system (MGOS). **b** Picture of an active MGOS operating in 100 ^o^C boiling water. **c** An active MGOS frozen in −20 ^o^C for 72 h. **d** One 2 × 2 MGOS array mounted on the abdominal skin of a living *Carassius auratus*. **e** Underwater wearable operational block diagram of the MGOS. **f** Picture of the fish wearing the 2 × 2 MGOS array underwater.

Since the harvesting unit is InGaN/GaN quantum well based, the device can harvest energy from short-wavelength light. Both power and information are delivered wirelessly through a 389.4-nm flashlight beam with a full width at half maximum of 10.3 nm. The harvesting unit efficiently converts light energy into electricity to directly turn on the LED. Figure 4a shows an active MGOS consisting of eight harvesting units and eight LEDs on a single chip. The generated electricity can power all eight LEDs when we shine light on the chip. Compared to the excited light source, monolithically integrated LED can be placed in the target position for accurate illumination and consequently, the illumination area will be smaller. Additionally, the wavelength of the emitted light can be adjusted as needed. Since the MGOS has no energy storage unit, LEDs turn off when the external light source is switched off, as demonstrated in Supplementary Movie 1. Since the interconnected metal wires are protected by the SiO_2_/TiO_2_ DBR layer, the MGOS is waterproof. Conventional wearable/implantable devices are usually encapsulated using polydimethylsiloxane. Compared with polydimethylsiloxane, both sapphire substrate and SiO_2_/TiO_2_ DBR films can sustain higher working temperature. To confirm it, the chip is put into a heat-proof beaker, and the water is heated using a heater. After maintaining the chip in 100 ^o^C water for 1 h, all eight LEDs turn on when the beam of the 389.4 nm flashlight strikes the chip, as show in Fig. 4b. Supplementary Movie 2 demonstrates practical operation of the MGOS in 100 ^o^C water. The boiling water produces air bubbles, and the MGOS can simultaneously achieve underwater energy harvesting and transfer through light. Moreover, the chip is put into water and frozen in a refrigerator with a temperature of −20 ^o^C. As a result, the chip is trapped inside ice. Since ice is transparent, the light beam can pass through it and transfer optical energy and signals to the harvesting unit. After freezing the chip in −20 ^o^C ice for 72 h, we use the 389.4 nm flashlight beam to activate the MGOS. Picture in Fig. 4c shows that all eight harvesting units can generate electricity from the incident light to directly cause all eight LEDs to turn on. Supplementary Movie 3 demonstrates practical operation of the MGOS frozen in −20 ^o^C ice for 72 h. Figure 4d shows that one 2 × 2 wearable MGOS array is mounted on the abdominal skin of a living *Carassius auratus*. The total weight was approximately 200 mg, whereas the weight of the 2 × 2 MGOS array was 170 mg. Figure 4e shows an underwater wearable operational block diagram. The flashlight beam passes through water and strikes the MGOS attached to the body of a freely-swimming fish, achieving real-time identification and tracking of moving animals and studying its natural behavior. As illustrated in Fig. 4f, fish wearing these chips can freely swim in a water tank, and underwater light energy transfer and communication are successfully realized by shining the flashlight beam on these chips. Supplementary Movie 4 demonstrates simultaneous operation of wireless light harvesting and emission for all the 2 × 2 MGOS arrays when the flashlight beam passes through the water and strikes these chips, which are attached to a living fish using waterproof tape. Such chip that monolithically integrates a III-nitride transmitter, receiver, and energy harvester will be developed as new medical devices, which can be implanted into a living mouse brain for exploring optogenetics. Both energy and information are wireless transferred via light, and the power generated by energy harvester will turn on monolithically integrated transmitter for optical stimulation, and monolithically integrated transmitter can also detect optical response from the mouse. The critical challenge for this chip is how to monolithically integrate energy storage, driver and receiver into a single GaN chip. Recently, without involving re-growth or post-growth doping, we successfully integrated GaN metal-oxide-semiconductor field effect transistors (MOSFETs), transmitter, waveguide, and receiver into a single chip^38^. The capability of integrating optoelectronics (transmitter, waveguide, receiver) with MOSFETs would inevitably open up new horizons for the GaN multicomponent systems with new interactive functions and multitasking devices.

## Conclusions

These results show that the MGOS can achieve wireless power transfer and communication through light, effectively addressing the challenge of underwater optoelectronics. The MGOS fabrication process is compatible with established GaN LED technologies and manufacturing tools, and a waterproof package is easily formed by depositing a SiO_2_/TiO_2_ DBR passivation layer to protect metal wires among devices, making it possible to monolithically integrate large numbers of devices into a single chip in a cost-effective manner. These simple, miniaturized, wearable MGOS architectures enable robust multifunctional operation without complicated external circuits, thereby providing intriguing features in a wide variety of underwater applications.

## Methods

### Fabrication

Epitaxial films consisting of unintentionally doped GaN (u-GaN), Si-doped n-GaN, InGaN/GaN multiple quantum wells, and Mg-doped p-GaN were grown on a 4 inch patterned sapphire substrate by metal-organic chemical vapor deposition. The mesa regions were defined by photolithography and etched at a depth of 1.4 μm to expose the n-GaN surface. Inductively coupled plasma (ICP) etching was performed with a mixture of Cl_2_ and BCl_3_. Deep ICP etching was further carried out to completely remove epitaxial films for device isolation. A 95 nm thick transparent indium tin oxide (ITO) current-spreading layer was deposited by sputtering, followed by rapid thermal annealing at 530 °C in a N_2_ atmosphere for 7 min. Subsequently, the ITO layer was patterned and etched away to expose the n-GaN surface using a mixture of HCl/FeCl_3_. Ni/Al/Ti/Pt/Ti/Pt/Au/Ti/Pt/Ti metal stacks were deposited on the n-GaN and ITO surfaces, followed by a metal lift-off process and rapid thermal annealing. A 1000 nm-thick SiO_2_ layer was deposited on the wafer by plasma-enhanced chemical vapor deposition. The electrode and bonding pad patterns were then photolithographically defined, and dry ICP etching was performed to etch the SiO_2_ layer away with a mixture of SF_6_, CHF_3_ and He. The Ni/Al/Ti/Pt/Ti/Pt/Au metal pads were then deposited by e-beam evaporation, followed by a metal lift-off process and rapid thermal annealing. The sapphire substrate was lapped and polished down to 200 μm, and the chips were finally diced by ultraviolet nanosecond laser micromachining. A waterproof device architecture formed after depositing a SiO_2_/TiO_2_ DBR on the device surface. The fab factory has performed reliability/long-term (stability) experiments for the devices.

### Reflectivity spectra characterization

The spectral characterizations of the TiO_2_/SiO_2_ DBR are performed by using the angle-resolved micro-reflection measurement system. A Bentham WLS100 quartz halogen lamp with a 200 μm-in-diameter fiber pigtail is used as the white light source. The incident light illuminates the sample with a circular light spot of ~20 μm in diameter, and the reflected light is then collected by another fiber pigtail connecting to an Ocean Optics USB2000 spectrometer. To observe the optical image of the measured sample, a CCD camera is used in the measurement system.

### EL and responsivity spectra characterization

The responsivity spectrum was measured using the Oriel IQE−200B (Newport Corp), in which a Xenon lamp is used as the light source, and a calibrated reference detector provides reliable and repeatable calibration. For the EL measurement, the emitted light was coupled into a 200 μm-in-diameter multimode fiber by a lens system and fed to an Ocean Optics HR4000 spectrometer for characterization.

### Simultaneous energy transfer and communication through light

A 405-nm laser pulses its light with encoded-information to illuminate on the harvesting units, thereby achieving wirelessly energy transfer and communication simultaneously. An external Hamamatsu C12702-11 photodiode module detects the spatial light emission from monolithically integrated LED to convert the photons back into electrons through the micro-transmittance setup. The received signals are directly sent to an Agilent DSOS604A digital storage oscilloscope for characterization.

### Experimental model and subject details

*Carassius auratus*, 18–19 cm in body length were used in this study. The fish were kept in a water tank at room temperature. One 2 × 2 MGOS array with a total weight of 200 mg was mounted on the abdominal skin of a living Carassius auratus using waterproof tape. The flashlight beam passes through water and strikes the MGOS attached to the body of a freely-swimming fish, achieving real-time identification and tracking of moving animals and studying its natural behavior. The experiment strictly abides by the rules of animal experiments. The experiment mainly focused on observing the behavior of fish, and did not bring physical harm to fish. After the experiment, the fish were released in the school’s lake on January 15, 2022.

All the experiments in *Carassius auratus* were approved by the Animal Ethics and Welfare Committee of Nanjing University of Posts and telecommunications and were in line with animal protection, animal welfare and ethical principles, as well as the relevant provisions of the national experimental animal welfare.

### Reporting summary

Further information on research design is available in the Nature Research Reporting Summary linked to this article.

### Supplementary information

Supplementary Information

Description of Additional Supplementary Files

Supplementary Movie 1

Supplementary Movie 2

Supplementary Movie 3

Supplementary Movie 4

Supplementary Data 1

Supplementary Data 2

Reporting Summary

## Data Availability

The data that support the findings of this study are available from the corresponding author upon reasonable request. Underlying data for the main manuscript figures is included as an excel file in source data.
