# Supplementary material for: Wireless light energy harvesting and communication in a waterproof GaN optoelectronic system
Source: Commun Eng. 2022 Jul 7;1:16. doi: 10.1038/s44172-022-00016-5 (PMC10956059; doi:10.1038/s44172-022-00016-5)
Supplement: Supplementary file 1 — Supplementary Information [file 44172_2022_16_MOESM1_ESM.pdf]

Supplementary Information

Wireless light energy harvesting and communication in a waterproof GaN optoelectronic system

Xumin Gao[1,2], Pengzhan Liu[1,2], Qingxi Yin[1,2], Hao Wang[1], Jianwei Fu[1], Fangren Hu[1], Yuan Jiang[1], Hongbo Zhu[1], Yongjin Wang[1,*]

[1]*Grünberg Research Centre, Nanjing University of Posts and Telecommunications, Nanjing 210003, China*

[2] *Authors contributed equally*

* *Corresponding author: wangyj@njupt.edu.cn*

Supplementary Figures

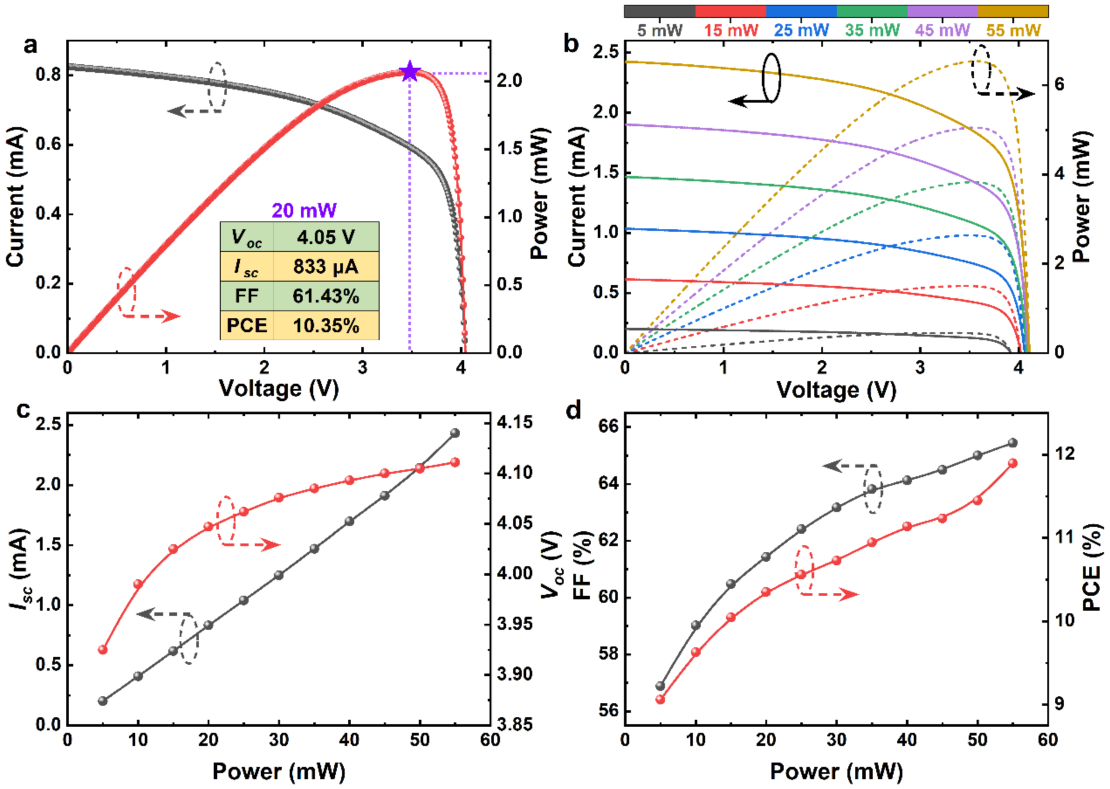

Supplementary Figure 1 The energy harvesting performance. (a) Room-temperature current-voltage (I-V) and power-voltage (P-V) characteristics of the individual harvesting unit under 20 mW laser irradiation. V_{oc}, open-circuit voltage; I_{sc}, short-circuit current; FF, filling factor; PCE, photon conversion efficiency. (b) I-V and P-V curves of the individual harvesting unit under different power levels of laser. The power dependence of (c) V_{oc}, I_{sc} and (d) FF, PCE as a function of laser power ranging from 5 mW to 55 mW.

The energy harvesting performance was experimentally characterized. Supplementary Figure 1(a) shows the typical current-voltage (I-V) and power versus voltage (P-V) characteristics of the individual harvesting unit under an incident laser centering at 405 nm with power of 20 mW. The measured open-circuit voltage (V_{oc}), short-circuit current (I_{sc}) and filling factor (FF) of the unit are 4.05 V, 833 μA, and 61.43%, respectively, corresponding to the photon conversion efficiency (PCE) of 10.35%. The incident light intensity dependence of the key parameters for the harvesting unit is also investigated. Supplementary Figure 1(b) shows the I-V and P-V curves of the harvesting unit under different power levels up to 55 mW. The curves of V_{oc} and I_{sc} as functions of irradiation power are illustrated in Supplementary Fig. 1(c). The I_{sc} increases linearly with laser power at a slope of about 42.2 μA/mW since the

number of charge carrier generated is directly proportional to the number of photons absorbed. The V_{oc} is logarithmically dependent on the irradiation power. The value of V_{oc} increases from 3.95 V to 4.12 V, and the FF value increases from 56.89% to 65.44% when the irradiation power increases from 5 mW to 55 mW, as shown in Supplementary Fig. 1(d). Compared with 5 mW illumination, the measured V_{oc} and FF increase by 4.3% and 15%, respectively, which corresponds to an enhancement of PCE by approximately 31.1%.

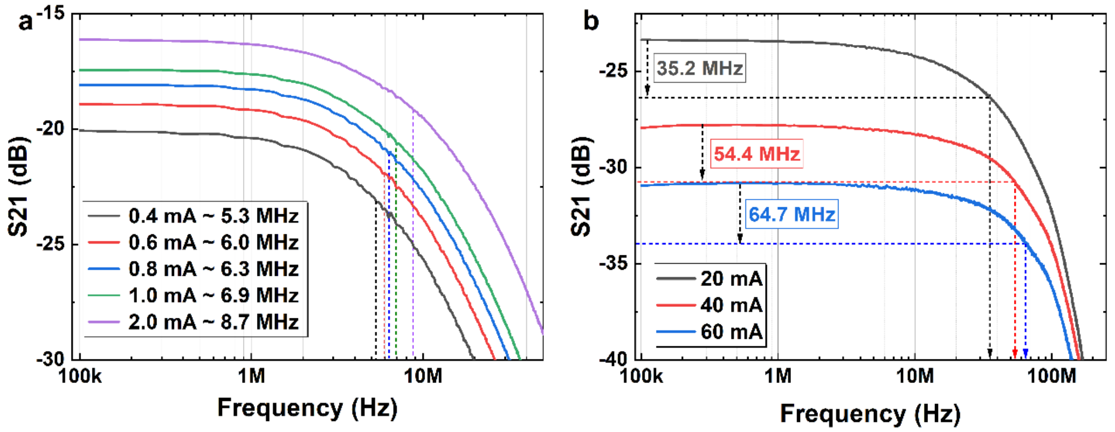

Supplementary Figure 2 The frequency response of the light emitting diode (LED). S21 parameters of LED under various injection current: (a) Current injection from 0.4 mA to 2.0 mA; (b) Current injection from 20 mA to 60 mA.

The frequency response of the light emitting diode (LED) in the range from 100 kHz to 200 MHz is measured using a network analyzer (NA, Agilent E5080A). The input signal of generated from the NA is combined with the direct current generated from source meter (Keithley 2636B) to drive the LED. The emission light of LED is then focused into a photodetector (Hamamatsu C12702-11). As shown in Supplementary Fig. 2(a), at the injection current level of 0.4 mA, LED has a -3dB bandwidth at 5.3 MHz. With increasing the driven current to 2 mA, the bandwidth increases to 8.7 MHz. Supplementary Figure 2(b) illustrates that the bandwidth of LED can reach to 64.7 MHz when the driven current is augment to 60 mA.

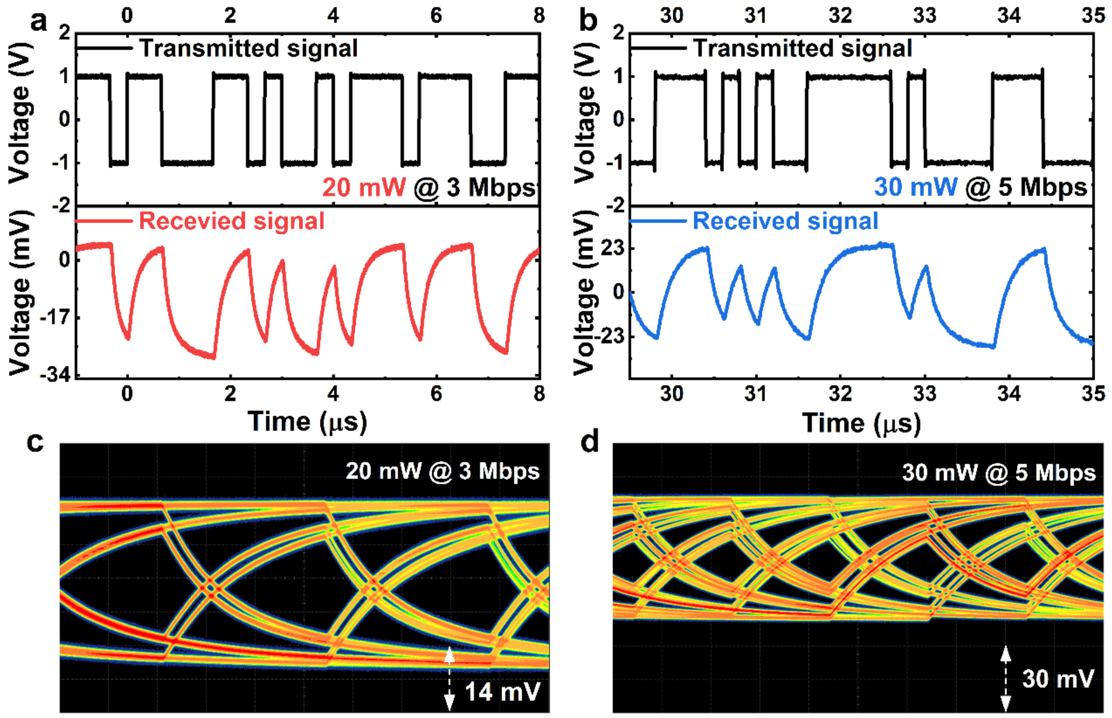

Supplementary Figure 3 The performance of the individual photovoltaic cell unit in the monolithic GaN optoelectronic system (MGOS). Comparison between transmitted and received pseudo-random binary sequence signals at transmission rate of (a) 3 Mbps under irradiation power of 20 mW; (b) 5 Mbps under irradiation power of 30 mW for the photovoltaic cell unit; (c) and(d): the corresponding eye diagrams of (a) and (b), respectively.

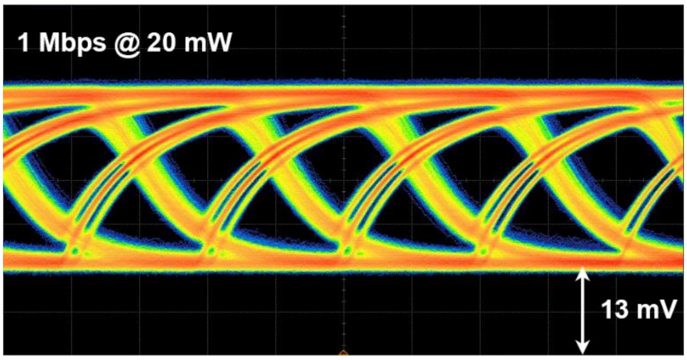

Supplementary Figure 4 The corresponding eye diagram for the quoted rate of Figure 3(f) in the manuscript.

A higher transmission rate is adopted to evaluate the impact of individual photovoltaic cell unit on the monolithic GaN optoelectronic system (MGOS). The experimental condition is in line with that in Figure 3(f) of the manuscript. As shown in Supplementary Fig. 3(a) compares the transmitted and received pseudo-random binary sequence signals at a communication rate of 3 Mbps with the same irradiation

power of 20 mW, and the corresponding eye diagram is shown in Supplementary Fig. 3(c). Increasing the irradiation power level can lead to a higher transmission rate. Supplementary Figures 3(b) and 3(d) show the measured pseudo-random binary sequence signals and eye diagram at a transmission rate of 5 Mbps with the irradiation power of 30 mW, suggesting that the photovoltaic cell can sustain the MGOS's performance. Supplementary Figure 4 shows the eye diagram for the quoted data rate of Figure 3(f) in the manuscript.
