# Supplementary material for: Wireless light energy harvesting and communication in a waterproof GaN optoelectronic system
Source: Commun Eng. 2022 Jul 7;1:16. doi: 10.1038/s44172-022-00016-5 (PMC10956059; doi:10.1038/s44172-022-00016-5)
Supplement: Supplementary file 2 — Description of Additional Supplementary Files [file 44172_2022_16_MOESM2_ESM.docx]

Description of Additional Supplementary Files

**File name:** Supplementary Movie 1

**Description:** Using external light to activate the LEDs for illumination and relaying light information.

**File name:** Supplementary Movie 2

**Description:** Practical operation of the waterproof MGOS in boiling water.

**File name:** Supplementary Movie 3

**Description:** Practical operation of the waterproof MGOS in ice.

**File name:** Supplementary Movie 4

**Description:** The MGOS chips simultaneous conduct wireless energy harvesting and light communication emission when attached to living fish freely swimming in a water tank.

**File name:** Supplementary Data 1

**Description:** Source data underlying the Figure 2a to Figure 2f in the manuscript.

**File name:** Supplementary Data 2

**Description:** Source data underlying the Figure 3b to Figure 3f in the manuscript.
