# Supplementary material for: Wireless light energy harvesting and communication in a waterproof GaN optoelectronic system
Source: Commun Eng. 2022 Jul 7;1:16. doi: 10.1038/s44172-022-00016-5 (PMC10956059; doi:10.1038/s44172-022-00016-5)
Supplement: Supplementary file 9 — Reporting Summary [file 44172_2022_16_MOESM9_ESM.pdf]

Corresponding author(s): Yongjin Wang

Last updated by author(s): Jun 23, 2022

Reporting Summary

Nature Research wishes to improve the reproducibility of the work that we publish. This form provides structure for consistency and transparency in reporting. For further information on Nature Research policies, see our Editorial Policies and the Editorial Policy Checklist.

Statistics

For all statistical analyses, confirm that the following items are present in the figure legend, table legend, main text, or Methods section.

n/a	Confirmed	
☒	☐	The exact sample size (n) for each experimental group/condition, given as a discrete number and unit of measurement
☒	☐	A statement on whether measurements were taken from distinct samples or whether the same sample was measured repeatedly
☒	☐	The statistical test(s) used AND whether they are one- or two-sided<br>*Only common tests should be described solely by name; describe more complex techniques in the Methods section.*
☒	☐	A description of all covariates tested
☒	☐	A description of any assumptions or corrections, such as tests of normality and adjustment for multiple comparisons
☒	☐	A full description of the statistical parameters including central tendency (e.g. means) or other basic estimates (e.g. regression coefficient) AND variation (e.g. standard deviation) or associated estimates of uncertainty (e.g. confidence intervals)
☒	☐	For null hypothesis testing, the test statistic (e.g. F, t, r) with confidence intervals, effect sizes, degrees of freedom and P value noted<br>*Give P values as exact values whenever suitable.*
☒	☐	For Bayesian analysis, information on the choice of priors and Markov chain Monte Carlo settings
☒	☐	For hierarchical and complex designs, identification of the appropriate level for tests and full reporting of outcomes
☒	☐	Estimates of effect sizes (e.g. Cohen's d, Pearson's r), indicating how they were calculated

Our web collection on statistics for biologists contains articles on many of the points above.

Software and code

Policy information about availability of computer code

Data collection	No software was used.
Data analysis	No software was used.

For manuscripts utilizing custom algorithms or software that are central to the research but not yet described in published literature, software must be made available to editors and reviewers. We strongly encourage code deposition in a community repository (e.g. GitHub). See the Nature Research guidelines for submitting code & software for further information.

Data

Policy information about availability of data

All manuscripts must include a data availability statement. This statement should provide the following information, where applicable:

- Accession codes, unique identifiers, or web links for publicly available datasets
- A list of figures that have associated raw data
- A description of any restrictions on data availability

The data that support the findings of this study are available from the corresponding author upon reasonable request. Underlying data for the main manuscript figures is included as an excel file in source data.

Field-specific reporting

Please select the one below that is the best fit for your research. If you are not sure, read the appropriate sections before making your selection.

☒ Life sciences ☐ Behavioural & social sciences ☐ Ecological, evolutionary & environmental sciences

For a reference copy of the document with all sections, see nature.com/documents/nr-reporting-summary-flat.pdf

Life sciences study design

All studies must disclose on these points even when the disclosure is negative.

Sample size	There was no sample-size calculation performed. The findings in the manuscript mainly focused on the performance of the waterproof monolithic GaN optoelectronic chip that integrates an energy harvester, LED and Bragg reflector. The units can transform external light into electrical energy and directly activate the integrated LEDs even when attached to freely swimming Carassius auratus fish. The chips were fabricated on a 4-inch epitaxial wafer. We have adopted dozens of chips to perform the experiments in the boiling water and ice, as well as the MGOS chips to form the 2×2 arrays attached to the fish to make sure the reproducibility of the experimental findings in the manuscript. The performance of the chips remained stable in the multiple experiments.
Data exclusions	There were no data excluded from the analyses.
Replication	We have repeated several sets of experiments to ensure the reproducibility of the experimental findings in the manuscript. The chips operated in boiling water and ice, all of which numbered more than 15, were randomly selected from 4-inch epitaxial wafers. We also randomly allocated MGOS chips to form multiple 2×2 arrays that were attached to 2 fish for the experiments. All experimental results remain consistent and support the conclusions in the manuscript.
Randomization	The MGOS chips used in the experiment were allocated randomly to form 2×2 arrays that attached to the fish, as well as the chips operated in boiling water and ice.
Blinding	The investigators were blinded to group allocation during data collection and analysis.

Reporting for specific materials, systems and methods

We require information from authors about some types of materials, experimental systems and methods used in many studies. Here, indicate whether each material, system or method listed is relevant to your study. If you are not sure if a list item applies to your research, read the appropriate section before selecting a response.

Materials & experimental systems

n/a	Involved in the study	
☒	☐	Antibodies
☒	☐	Eukaryotic cell lines
☒	☐	Palaeontology and archaeology
☐	☒	Animals and other organisms
☒	☐	Human research participants
☒	☐	Clinical data
☒	☐	Dual use research of concern

Methods

n/a	Involved in the study	
☒	☐	ChIP-seq
☒	☐	Flow cytometry
☒	☐	MRI-based neuroimaging

Animals and other organisms

Policy information about studies involving animals; ARRIVE guidelines recommended for reporting animal research

Laboratory animals	The study did not involve laboratory animals.
Wild animals	The fish used in the experiment were Carassius auratus, which were caught using a fishing net from the Yanjing Lake in the Sanpailou Campus of Nanjing University of Posts and Telecommunications.There were two fish were caught, transported by a water tank. After the study, the fish were released back to the Yanjing Lake transported by water tank on January 15, 2022.
Field-collected samples	The study did not involve samples collected from the field.
Ethics oversight	The Animal Ethics and Welfare Committee of Nanjing University of Posts and Telecommunications.

Note that full information on the approval of the study protocol must also be provided in the manuscript.
